# Genome data of *Stenotrophomonas maltophilia* DF07 collected from polluted river sediment reveals an opportunistic pathogen and a potential antibiotic reservoir

**DOI:** 10.1016/j.dib.2019.104137

**Published:** 2019-06-11

**Authors:** Rupa Iyer, Ashish Damania, Brian Iken

**Affiliations:** a Center for Life Sciences Technology, Engineering Technology, University of Houston, Houston, USA; b Department of Pediatrics-Tropical Medicine, Baylor College of Medicine, Houston, USA

**Keywords:** Antibiotic resistance, Genome, Plasmid, San Jacinto River, *Stenotrophomonas maltophilia*

## Abstract

*Stenotrophomonas maltophilia* DF07 is a gram negative bacterium isolated from polluted San Jacinto River sediment near Moncrief Park in Channelview, Texas. The genome of strain DF07 (chromosome and plasmid) was compiled at the scaffold level and can be accessed through the National Center for Biotechnology Information database under accession NZ_NJGC00000000. The DF07 genome consists of a total of 4,801,842 bp encoding for approximately 4,351 functional proteins. Approximately 86 proteins are associated with broad-spectrum antibiotic resistance, 11 are associated with bacteriocin production, and a total of 17 proteins encode for an assortment of *Mycobacterium*-like virulence and invasion operons. *S. maltophilia* DF07 is genetically similar to the nosocomial *S. maltophilia* strain AU12-09, but also harbors an unusually large plasmid that encodes for over 150 proteins of unknown function. Taken together, this strain is potentially an important antibiotic reservoir and its origin within a recreational park merits further study of the area.

**Table:** Specifications Table

Subject area	Microbiology
More specific subject area	Microbial genetics
Type of data	Figures, DNA sequence
How data was acquired	DNA sequencing: Illumina Miseq Bioinformatics: NCBI Prokaryotic Genomes Automatic Annotation Pipeline (PGAAP), the RAST web server
Data format	Raw and analyzed
Experimental factors	Genomic DNA from pure microbial culture
Experimental features	Microbial sample was isolated from polluted sediment along San Jacinto River and whole genome sequenced using Illumina MiSeq technology
Data source location	Polluted sediment/soil USA: Texas, Channelview, Moncrief Park (29.805,619, −95.095,543)
Data accessibility	Strain data is uploaded to National Center for Biotechnology Information database under accession NZ_NJGC00000000. Direct link to data: https://www.ncbi.nlm.nih.gov/Traces/wgs/NJGC01?display=proteins&page=1
Related research article	L. Zhang, M. Morrison, P.O. Cuív, P. Evans, C.M. Rickard. Genome sequence of *Stenotrophomonas maltophilia* strain AU12-09, isolated from an intravascular catheter. Genome Announc., 1 (2013), e00195-13 [1].

**Table:** 

**Value of the data** • The genome data of *S. maltophilia* DF07 highlights the presence of an unusually large plasmid. *S. maltophilia* DF07 therefore provides insight into horizontal gene transfer and possibly the spread of antibiotic resistance and virulence determinants across the San Jacinto River in addition to other polluted waterways. • This genomic data expands our understanding of the potential for opportunistic pathogenicity in *S. maltophilia* isolates and their capacity to act as an antibiotic resistance reservoir. • The data presented in this brief can be used in antibiotic resistance comparisons between environmental and nosocomial (clinical) isolates of *S. maltophilia.*

## 1 Data

**Fig. 1:**
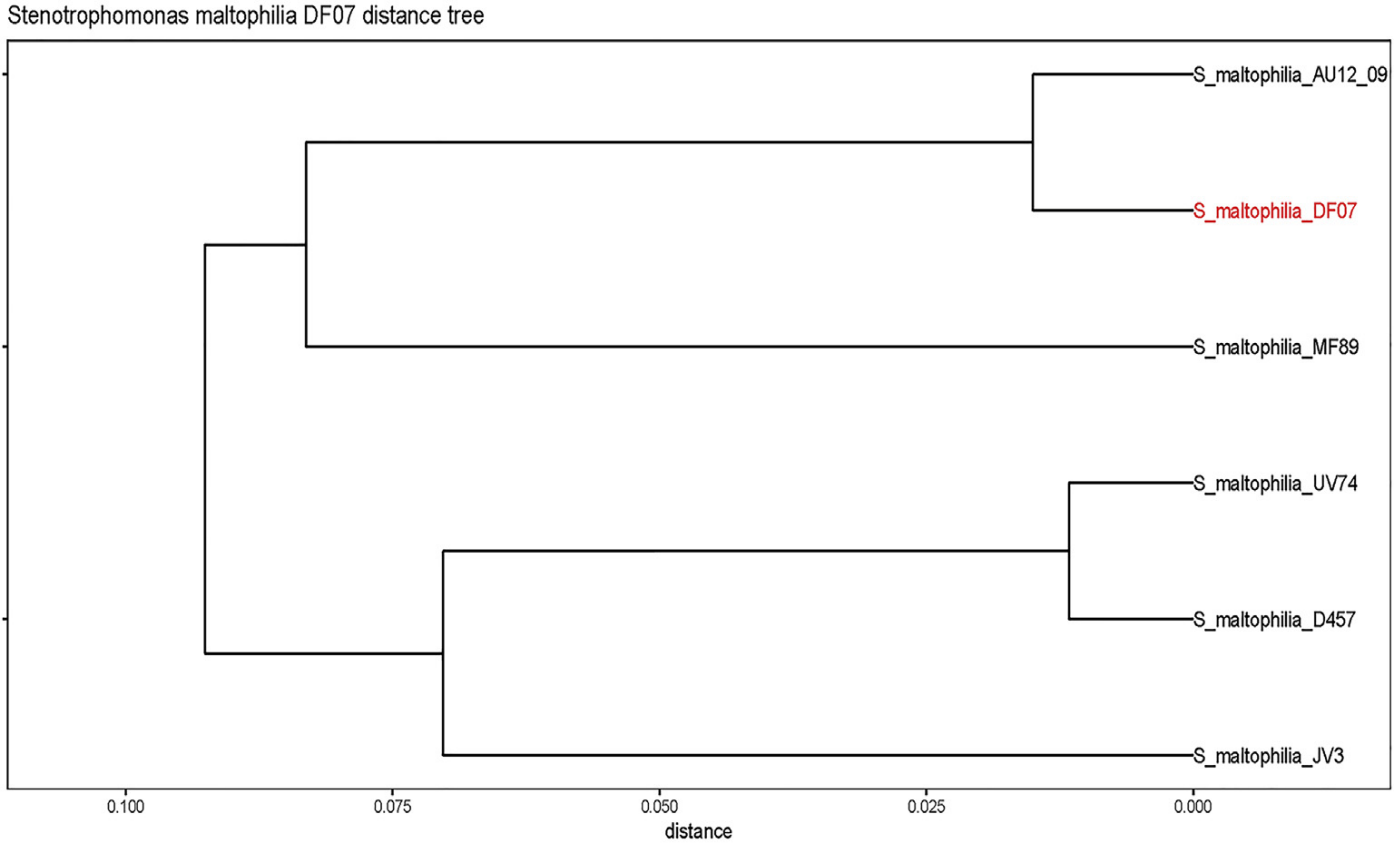
UPGMA dendogram of *S. maltophilia* DF07 and the top five similar bacterial genomes.

**Fig. 2:**
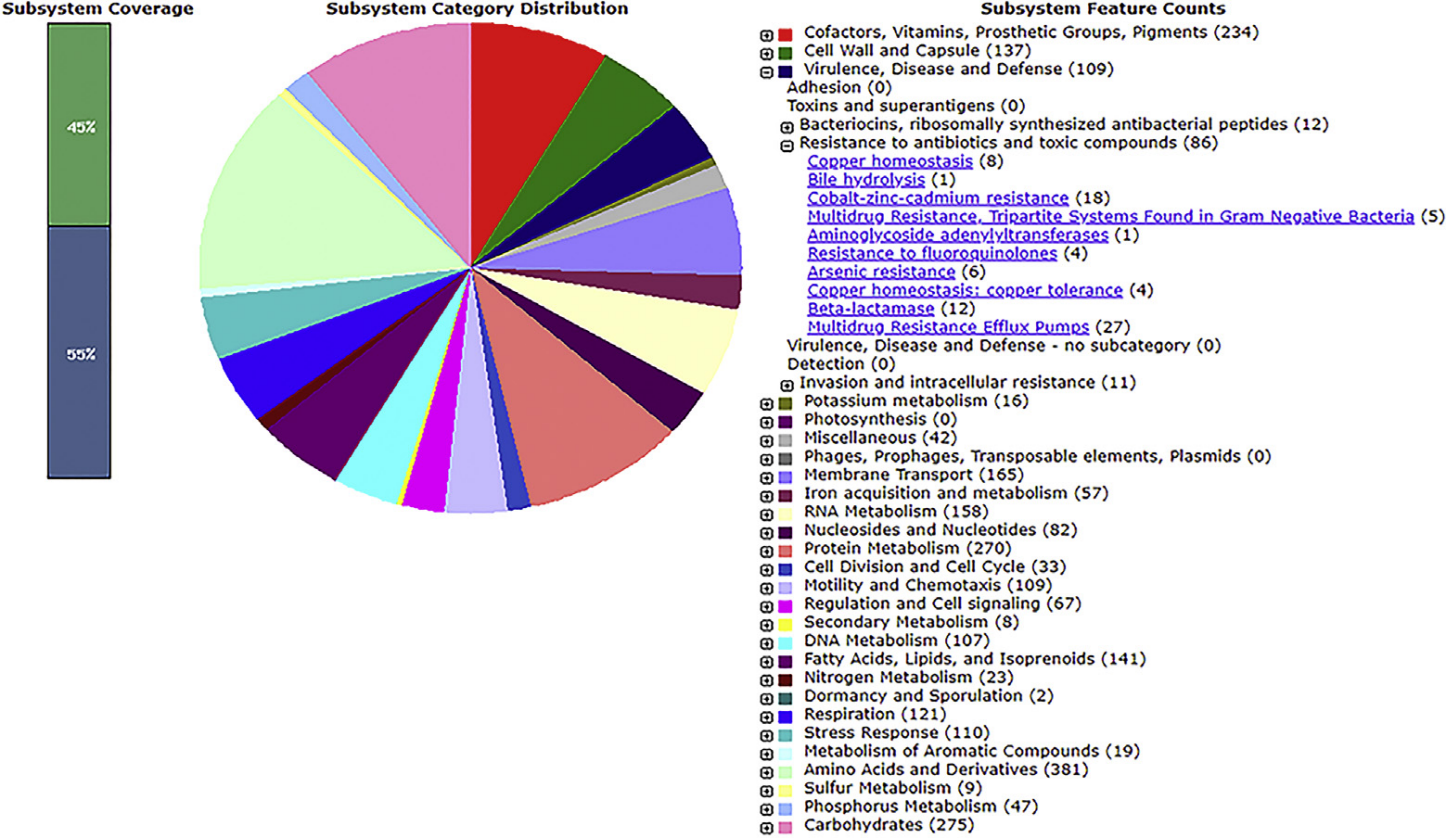
**Subsystem category distribution of major protein coding genes on the chromosome of *S. maltophilia* DF07 as annotated by the RAST annotation server.** The bar chart shows the subsystem coverage in percentage (the green bar corresponds to percentage of proteins identified in one of the listed subsystems). The pie chart shows the distribution of the 25 most abundant subsystem categories.

**Fig. 3:**
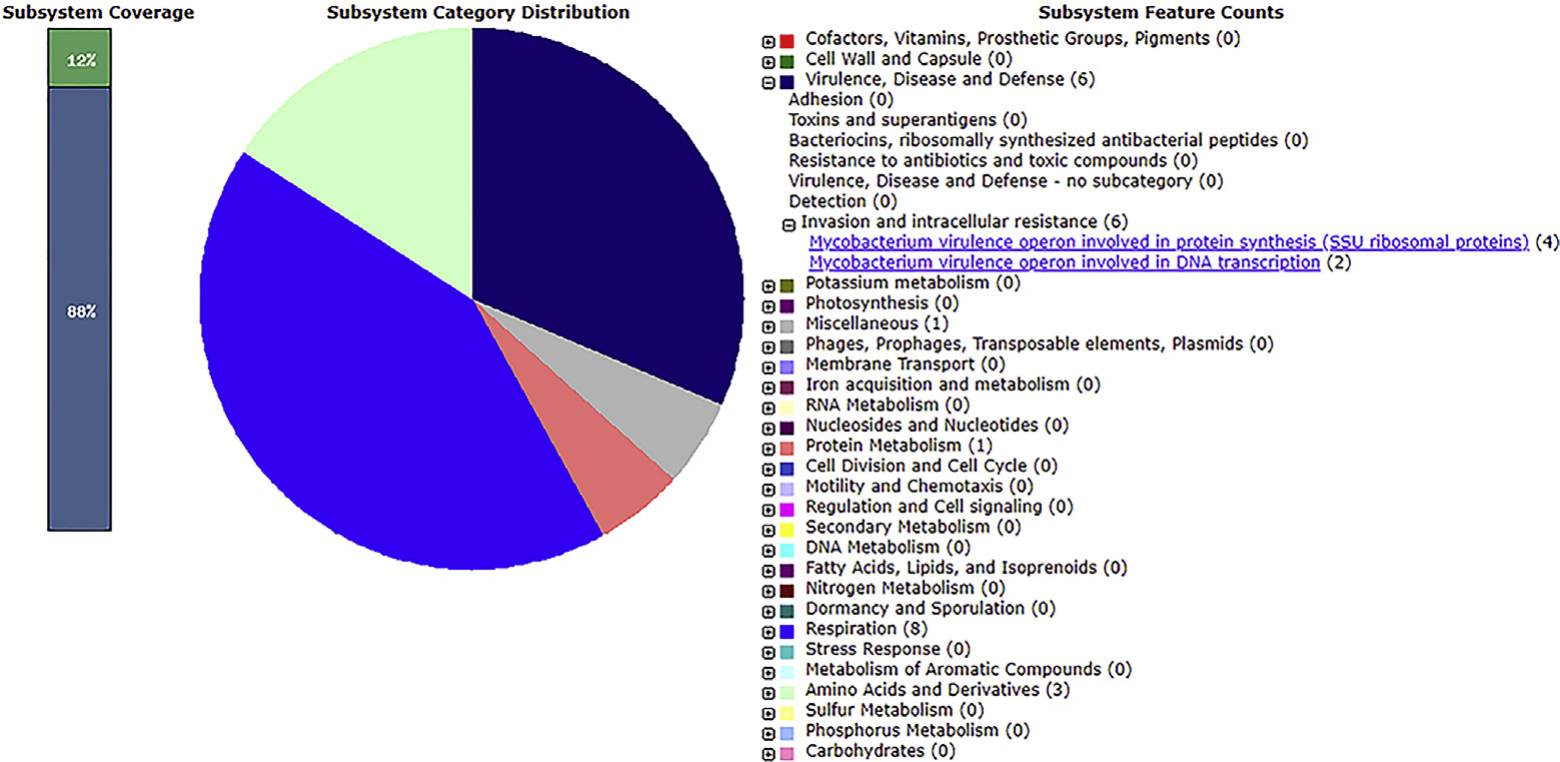
**Subsystem category distribution of major protein coding genes on the plasmid of *S. maltophilia* DF07 as annotated by the RAST annotation server.** The bar chart shows the subsystem coverage in percentage (the green bar corresponds to percentage of proteins identified in one of the listed subsystems). The pie chart shows the distribution of the 25 most abundant subsystem categories.

Sequence analysis identifies DF07 as a novel strain of *Stenotrophomonas maltophilia*, a member of the *Xanthomonadaceae* family from the *Gammaprotebacteria* class. This Gram-negative bacterium is ubiquitously distributed throughout both soil and aquatic environments. S. maltophilia is known to be an opportunistic human pathogen. According to MASH genome analysis, the closest relative of *S. maltophilia* DF07 is *S. maltophilia* AU12-09, a nosocomial isolate collected from a hospital intravascular catheter (Fig. 1) [1]. Genome annotation reveals that *S. maltophilia* DF07 possesses many of the antibiotic resistance determinants identified in strain AU12-09. These determinants include a complement of 12 β-lactamase enzymes and associated proteins, aminoglycoside inactivation enzymes, fluoroquinolones resistance proteins as well as 5 tripartite and 27 multidrug pump proteins related to antibiotic efflux (Fig. 2). Of particular note is the large bacterial plasmid found within the DF07 strain. This plasmid has a length of 209,390 bp and encodes for approximately 179 genes, many of which have unknown functions. *S. maltophilia* DF07 also encodes for several chromosomal and plasmid based *Mycobacterium*-like virulence and invasion operons (Fig. 3).

## 2 Experimental design, materials, and methods

### 2.1 Sample collection

Sediment was collected from the bottom of a 12 inch hole dug by the bank of the San Jacinto River alongside Moncrief Park in northern Channelview, Texas. Moncrief Park lies west of the now enclosed San Jacinto River Waste Pits, a submerged Superfund site once used for dumping of paper mill waste.

### 2.2 Sample screening

Carbon selective media was prepared as previously described in Iyer et al., 2016 [2]. A total of 5 mL of carbon selective media was used for initial sample inoculation. Dibenzofuran added at a final concentration of 100 μg/mL was used as a screening agent and potential carbon source. Subcultures were performed over five weeks before plating onto minimal agar plates supplemented with dibenzofuran.

### 2.3 Genomic DNA preparation

Plated colonies, yellow in coloration, were revitalized in 5 mL Luria-Bertani medium and grown overnight. Total cellular DNA of the overnight culture was then extracted using a Qiagen DNeasy Blood and Tissue kit.

### 2.4 Whole genome sequencing

Prepared sample DNA was shipped to Genewiz (South Plainfield, NJ) who performed Illumina MiSeq paired-end sequencing (Table 1).

**Table 1:** *S. maltophilia* DF07 genome statistics.

Assembly statistics	
platform	Illumina MiSeq (2*250) paired end
genome size(bp)	4,801,355
number of contigs	154 (chromosome) + 6 (plasmid)
average coverage	223.26x
Annotation statistics	
GC content	66.40 (chromosome) + 62.20 (plasmid)
total genes	4,522
coding genes	4,351
RNAs	87

### 2.5 Genome annotation

Raw sequence data was first quality checked in Fastqc [3] and poor reads filtered out using BBTools [4]. Good sequence reads were then assembled with the Spades 3.10 program [5]. Annotation was performed both through the NCBI Prokaryotic Genomes Automatic Annotation Pipeline (http://www.ncbi.nlm.nih.gov/genomes/static/Pipeline.html) and RAST server (See Table 1) [6], [7].

### 2.6 Phylogeny analysis

The Mash program was used first for species identification, then to map the five closest bacterial hits based on their Mash distances to the sample strain using the Mash sketch database for RefSeq release 70 (k-mer size = 21, sketch size = 1000) [8]. The file was then imported into R and the Ggdendrogram [9], [10] package used to create a phylogenetic tree (See Fig. 1).

## References

[1] Zhang L., Morrison M., Cuív P.O., Evans P., Rickard C.M. (2013). Genome sequence of *Stenotrophomonas maltophilia* strain AU12-09, isolated from an intravascular catheter. Genome Announc..

[2] Iyer R., Aggarwal J., Iken B. (2017). Genome of *Pseudomonas nitroreducen*s DF05 from dioxin contaminated sediment downstream of the San Jacinto River waste pits reveals a broad array of aromatic degradation gene determinants. Genomics data.

[3] Bioinformatics B. (2011). FastQC a Quality Control Tool for High Throughput Sequence Data. http://www.bioinformatics.babraham.ac.uk/projects/fastqc/.

[4] Bushnell B. (2016). BBMap Short Read Aligner. http://sourceforge.net/projects/bbmap.

[5] Bankevich A., Nurk S., Antipov D., Gurevich A.A., Dvorkin M., Kulikov A.S., Lesin V.M., Nikolenko S.I., Pham S., Prjibelski A.D., Pyshkin A.V., Sirotkin A.V., Vyahhi N., Tesler G., Alekseyev M.A., Pevzner P.A. (2012). SPAdes: a new genome assembly algorithm and its applications to single-cell sequencing. J. Comput. Biol..

[6] Tatusova T., DiCuccio M., Badretdin A., Chetvernin V., Ciufo S., Li W. (2013). Prokaryotic Genome Annotation Pipeline. The NCBI Handbook.

[7] Aziz R.K., Bartels D., Best A.A., DeJongh M., Disz T., Edwards R.A., Formsma K., Gerdes S., Glass E.M., Kubal M., Meyer F. (2008). The RAST server: rapid annotations using subsystems technology. BMC Genomics.

[8] Ondov B.D., Treangen T.J., Melsted P., Mallonee A.B., Bergman N.H., Koren S., Phillippy A.M. (2016). Mash: fast genome and metagenome distance estimation using MinHash. Genome Biol..

[9] Wickham H., Hester J., Francois R. (2016). Readr: Read Tabular Data. https://CRAN.Rproject.org/package=readr.

[10] de Vries A., Ripley B.D. (2016). Ggdendro: Create Dendrograms and Tree Diagrams Using ‘ggplot2’. R Package Version 0.1–20. https://CRAN.R-project.org/package=%20ggdendro.

